# Efficacy assessment in trials of complex and rare diseases: a comparison between the meta-analytic global statistical test and co-primary analysis

**DOI:** 10.3389/fmedt.2026.1819463

**Published:** 2026-06-19

**Authors:** Samuel P. Dickson, Abe Durrant, Caleb W. Dayley, Joshua R. Christensen, Craig H. Mallinckrodt, Suzanne B. Hendrix

**Affiliations:** Pentara Corporation, Millcreek, United States

**Keywords:** clinical trials, complex disease, co-primary endpoints, Global Statistical Test (GST), rare disease

## Abstract

**Introduction:**

Choice of the primary outcome can be problematic in 1) diseases with heterogeneous signs and symptoms, 2) trials of disease-modifying treatments (DMTs) that are expected to affect all aspects of the disease, 3) in complex and rare diseases with minimal data on clinical trial outcomes. In such situations, a single outcome measuring a single domain of disease will rarely suffice as a primary outcome. To address this issue, regulatory bodies often suggest co-primary endpoints or require efficacy on both primary and key secondary endpoints for confirmatory trials, to ensure that at least two different domains of disease are affected by treatment. However, obtaining statistical significance on two outcomes is a much stricter requirement than on a single primary outcome. Global statistical tests (GST) combine multiple outcomes into a single score and could provide a viable alternative to the co-primary approach. Importantly for rare diseases, combining multiple assessments reduces the risk of selecting a poorly performing outcome simply because it has not been studied extensively.

**Methods:**

We conducted simulations to compare GST to single primary and co-primary endpoint approaches with two moderately or highly correlated outcomes with various effect sizes.

**Results:**

For scenarios with the same true effect size on both outcomes, the GST had greater power than single primary and co-primary approaches, regardless of the correlation level between outcomes. This was also true with different effect size combinations at the same correlation level. With an effect observed on one outcome only, GST was more likely to yield statistical significance than the co-primary approach. Unlike the co-primary approach, The GST yielded lower *p*-values in scenarios with lower correlation between the outcomes due to the independence of the information from each endpoint.

**Conclusions:**

Using GST as a prespecified endpoint is appropriate in trials where a clear primary endpoint has not been identified, sample sizes are insufficient to support multiple primary endpoints, and/or more comprehensive assessments across multiple endpoints are needed to fully evaluate outcomes.

## Introduction

Drug development is not a one-size-fits-all proposition. Development strategies, study designs, and analyses need to be tailored to each situation. One of the most important design choices is the primary endpoint. Drug development in complex and rare diseases can pose challenges that result in difficulty in selecting a single, primary endpoint. Additionally the inherently small sample sizes available for study of rare diseases results in individual endpoints that often lack sufficient precision to support robust decision-making.

In well-researched and well-understood diseases, convention has often settled on a single primary endpoint. In some cases, convention has settled on co-primary endpoints to simultaneously consider multiple disease domains. Typically, statistical significance on both endpoints is required to declare success (1, 2). The co-primary endpoint approach is conceptually simple, but a steep price is paid in terms of sample size and statistical power because obtaining significance on two outcomes is always a stricter requirement than significance on a single primary outcome.

Rare and complex diseases introduce additional complications when choosing a primary endpoint. Complex diseases have multiple domains that mark disease progression, making it difficult to capture all aspects of disease progression at all severities using a single outcome. In addition to often being complex, rare diseases will usually have limited data on outcomes—using either proven outcomes designed for other diseases but with only limited experience in the rare disease being studied or outcomes designed for the rare disease but considered unproven from the perspective of time and experience. Given the limited sample sizes available for rare diseases, it is usually implausible to anticipate clearly which outcomes will properly discern true treatment effects in clinical trials.

The Global Statistical Test (GST), first introduced in 1984, is a single endpoint that combines results across multiple endpoints into a single estimate, single confidence interval, and a single *p*-value. The outcome evaluated in a GST is conceptually an average of the effects across the endpoints included in the GST. As such, in the context of clinical trials in complex rare diseases, where the goal is to measure a treatment's effect across multiple domains, and the expected signal and noise are likely not well-established in the population of interest for the outcome measuring each domain of the disease, a GST will be less affected by the uncertainty or loss of power that would be expected using other strategies like co-primary endpoints, alternative primary endpoints, or hierarchical testing.

Co-primary endpoints have often been pushed for complex diseases to ensure that all domains of the disease are affected by the treatment. Co-primary endpoints require that all endpoints specified as primary achieve statistical significance before the trial can be considered a success, with the result being that type I error for these trials is unfairly controlled at a much stricter rate than standard. Type I error for a standard trial using a two-sided alpha of 0.05 is 0.025 (since success would typically only be declared in one direction). Conventionally, there has been no upward adjustment in alpha to ensure that trials with co-primary endpoints are not held to higher standards than a typical trial, yet in order for type I error to be controlled at the same level using co-primary endpoints, the endpoints would have to be correlated at 100% or the alpha for each endpoint would have to be increased. For instance, two endpoints correlated at 30% would need to use alpha = 0.219 to maintain type I error at 0.025. Co-primary endpoints are costly—especially for complex rare diseases that don't have patient populations large enough to overcome the alpha penalty.

Alternative primary endpoints allow for a declaration of success if any of the primary endpoints is significant, but this requires a multiplicity adjustment to maintain type I error at 0.025. The simplest and most conservative adjustment is a Bonferroni adjustment that tests at 2-sided alpha/k, where k is the number of primary endpoints. Other adjustments are less strict, but all are focused on whether individual endpoints from single domains achieve significance rather than incorporating evidence across the domains as an indication broad efficacy in the disease.

Hierarchical gatekeeping approaches are common in clinical trials of complex diseases. In this design, other outcomes are not tested for significance unless all outcomes higher in the hierarchy have also achieved significance. In addition to testing only one outcome at a time instead of all relevant to the disease, this strategy also fails to address uncertainty regarding the performance of outcomes in rare diseases.

The GST has similar benefits as other composite endpoints because it is the sum of more than one item or assessment. Clinician-derived composites, like CDR-SB (3, 4), ADAS-Cog (5), or SARA (6), are the sums of the scores of several item-level assessments assigned point values based on clinician experience and intuition. While neither clinician-derived composites nor GSTs are specifically optimized to detect disease progression, a GST can combine outcomes across disease domains and guarantee that each domain has been given a specified weight in the composite. Clinician-derived composites can only rely on the experience and intuition of the clinicians involved in deriving the composites and cannot easily be adapted to the specific needs of the disease being studied. A GST is also different from optimized composite scores like ADCOMS (7), the APCC (8), and cUHDRS (9), which use historic data to optimize the weighting of different items to find a total score that is optimally sensitive to disease progression. The data necessary to derive this type of composite is likely not sufficiently available for a rare disease. Clinician-derived scores gain acceptance through years of experience and familiarization in the clinic. Optimized composites gain credibility by drawing on an abundance of historic data. In the absence of years of experience of clinician-derived composites and the historic data used for optimal composites, a GST provides similar benefits, because it can draw evidence from multiple domains and combine that evidence into a single testable hypothesis. This hypothesis is well-suited to scenarios where it is not clear which endpoint is most important; and the averaging also reduces random variability and results in greater precision and potentially greater power than any individual endpoint. In this sense, a GST can play a similar role to diversified portfolios in the investment world. Though individual investments have a high degree of uncertainty and could lead to losses on their own, when multiple investments are combined to appropriately capture a larger sector of the economy, when that sector of the economy improves, so will the portfolio.

The purpose of the present investigation is to 1) compare and contrast the hypotheses addressed by GSTs, co-primary endpoints, and other testing strategies; 2) illustrate the synthesis of information via GSTs in relevant outcome scenarios; and, 3) compare the power and type I error rate for a GST comprising two outcomes vs. a single primary endpoint and a co-primary endpoint. The specific example provided is one that is relevant in rare diseases with a symptomatic treatment effect, where the possibility of washout allows researchers to maximize information from participants through a crossover design. It is beyond the scope of this paper to compare GST to every possible method of inference involving multiple endpoints.

## Methods

### Hypotheses

Conceptually, co-primary endpoints test an AND hypothesis, step-up and step-down multiple testing procedures test an OR hypothesis, GSTs test an AVERAGE hypothesis, and sequential tests evaluate conditional secondary hypotheses.

The hypotheses tested by a single primary, co-primary, and GST endpoint are specified below.

When two or more endpoints are combined into a GST, the test evaluates the hypothesis that the average effect of the treatment on the endpoints is 0. For example, if three endpoints are included in the GST, the null and alternative hypotheses are:

H₀: *δ*_123_ = 0, and Ha: *δ*_123_ > 0 for one-tailed testing and Ha: *δ*_123_ ≠ 0 for two-tailed testing, where *δ*_123_ is the average difference between treatments on endpoints 1, 2, and 3.

The null hypothesis for co-primary endpoints is H₀: *δ*₁ = 0 OR *δ*₂ = 0, and the alternative hypothesis is that Ha: *δ*₁ > 0 AND *δ*₂ > 0 for one-tailed testing or *δ*₁ ≠ 0 AND *δ*₂ ≠ 0 for two-tailed testing. If a step up or step down multiplicity control testing scheme is used (e.g., Holm or Hochberg), the null and alternative hypotheses for each endpoint are the usual H₀: *δ*₁ = 0 and Ha: *δ*₁ > 0 for one-tailed testing or *δ*₁ ≠ 0 for two-tailed testing. However, it is also useful to consider the hypotheses in light of all the endpoints included in the testing scheme. With three endpoints, the null hypothesis is H₀: *δ*₁ = 0, *δ*₂ = 0, and *δ*_3_ = 0, and the alternative hypothesis is Ha: *δ*₁ > 0 OR *δ*_2_ > 0 OR *δ*_3_ > 0 for one-tailed testing (or ≠ 0 for two-tailed testing).

In sequential testing of a single primary endpoint and a series of secondary endpoints, the null and alternative hypotheses are specific to each individual endpoint (not joint endpoints), conditional on rejection of preceding hypotheses.

### GST considerations

As an average across endpoints, GSTs can be an objective way to aggregate information across endpoints to assess the total level of evidence for a treatment effect (10). An important aspect of aggregating the information is to consider how independent or related are the component outcomes included in the GST. Commonly used versions of the GST account for overlapping information (i.e., correlation) between endpoints, thereby providing a valid estimate of the total amount of information in the GST. For example, in healthy individuals, the correlation coefficient between forced expiratory volume in 1 s (FEV1) and forced vital capacity (FVC) can be as high as 0.96 (11). Combing these two endpoints into a GST would add very little information compared with either endpoint alone. However, correlations between endpoints measuring cognition and function in Alzheimer's disease are typically < 0.5 (12, 13), thereby suggesting much is gained by combining multiple assessments.

The original GST (14) was non-parametric and used a summed rank score across endpoints, resulting in a range of scores that increased with additional endpoints and with larger sample sizes. Parametric versions have also been developed, based on ordinary least squares and generalized least squares (14, 15). In these parametric approaches, z-scores are calculated for each patient and outcome at each visit. The visit-wise z-scores are averaged for each patient and the average z score for each patient at each visit is fit as the dependent variable in an analysis that would be suitable for the component outcomes.

A meta-analytic GST (MGST) has also been proposed (16) and is similar to the OLS/GLS method proposed by O'Brien, in which evidence is combined by calculating the joint probability of observing two test statistics simultaneously after accounting for the average correlation between each pair of endpoints. This approach allows the integration of results from different analytic approaches (e.g., response rate, mean change, or survival time), provided a correlation or another measure of overlapping information is established. The two GST approaches are similar in concept, with the MGST approach analyzing individual outcomes and combining results whereas the OLS/GLS approach averages outcomes first then analyzes the average outcome. The MGST approach was used here for convenience of illustration and will hence be referred to simply as GST.

The formula for the GST follows a standard normal distribution for samples of sufficient size:

$$GST=\frac{\bar{z}}{\{[1+(k-1)\rho ]/k\}{\;}^{1/2}}∼N(0,\;1),$$

where $\bar{z}$ is the average of the z-score test statistics from the hypothesis test for each endpoint after ensuring that the directionality of all endpoints is the same, ρ is the average correlation across each pair of outcomes, and *k* is the number of outcomes being combined. The z-scores can be replaced with t-scores for sample sizes that are small (< ∼15 per arm), resulting in a t-distributed test statistic (which is equivalent to the OLS/GLS).

While the example scenario in this paper is simplified to highlight specific clinical trial concepts for rare diseases, it is important in practice to verify statistical assumptions for every endpoint. It is also important to note that the subject-level GSTs considered here require continuous outcomes.

The one-sided *p*-value for the GST is calculated as the cumulative probability associated with the test statistic in the direction of the alternative hypothesis. In contrast, co-primary analysis involves a separate assessment of statistical significance on each endpoint, and evidence of efficacy is typically based on statistical significance on both co-primary endpoints.

### Examples of GST outcomes illustrated for various clinical trial scenarios

Eight hypothetical examples are shown with results from a clinical trial including three endpoints, with a correlation of 0.40 between each pair of endpoints. In each example, the first endpoint is statistically significant with *p* = 0.049, two-sided. Endpoints 2 and 3 have equal and non-significant *p*-values. The various combinations illustrate how results of various robustness combine for a single inference.

### Simulation study

In addition to the eight hypothetical examples, to compare GST analysis to co-primary analysis, a simulation was performed to mimic a theoretical clinical trial that evaluates an investigational therapy in patients with a rare, complex, and heterogenous disease. The simulation study is designed following the “ADEMP” framework (17). The setting of a crossover design was chosen so that results could be evaluated in the “adequately powered” crossover data and in the “under powered” period I of the study analyzed as a parallel group design. The hypothetical simulated clinical trial data were based on a two-treatment, two-period, two-sequence cross-over design. Two endpoints were simulated for each patient at each time point, with 16 participants per sequence (total *N* = 32). No carryover effects were simulated. Each patient had a baseline and a post-baseline assessment for both endpoints in each period. For each outcome, correlation between observations of the same patient across the two phases (within a treatment sequence) was assumed to be 0.5.

Several scenarios were simulated, in the null hypothesis scenario, the effect size for each co-primary outcome = 0 and in other scenarios the treatment effect corresponded to Cohen's *d* values (standardized treatment differences) of 0.3, 0.4, and 0.5. Within each combination of effect sizes, two levels of correlation between outcomes were used: 0.9 (strong) and 0.4 (moderate). For each effect size – correlation scenario data were analyzed from period 1 only as an underpowered parallel group study and using the full cross-over data from periods 1 and 2 in an adequately powered scenario. Overall, this resulted in 16 scenarios simulated, as a 4 × 2 × 2 factorial, with 4 levels of treatment effect, two levels of correlation, and two levels of amount of information.

For each scenario, we generated 10,000 simulated data sets.

The data were simulated and the analysis was conducted using R version 4.3 or later and the mvtnorm package (18). The two phases of the study were simulated with subjects evenly split into the different treatment sequences, placebo-to-active and active-to-placebo The subject score on each of the outcomes was simulated from a multivariate normal distribution with means of 0 for the simulated placebo subjects and means equal to the effect size in the given scenario for the simulated treatment subjects. The covariance matrix included a standard deviation of 1 with a correlation between outcomes dependent on the given scenario (0.9 or 0.4). The correlation between subjects was also factored into the covariance matrix with the subject level correlation between the two phases set at 0.5.

### Data analysis

Data obtained using simulation procedures were analyzed for a single primary endpoint, for the co-primary endpoints, and for the GST endpoint using the meta-GST approach, with a 2-sided alpha of 0.10. The main analysis considered the entire treatment sequence. We also conducted an auxiliary analysis, which only considered the first treatment period in each sequence.

In the analysis of the full cross-over data, test statistics and *p*-values were obtained from a mixed-effects model with repeated measures (MMRM), the standard analytic approach for trials with longitudinal continuous outcomes, then used it to derive the GST estimates via the formula above. The MMRM used to obtain the T-ratios included treatment (not treatment sequence) as a fixed effect and random intercepts by participant.

In the analysis of period 1 only, test statistics for the single primary endpoint and for the co-primary analysis were calculated via t-test. The GST was derived using the formula above. For each scenario, we calculated the power for detecting a treatment effect as well as the adjustment needed for the type I error, based on the percentage of simulated data sets in which the result was statistically significant.

## Results

### Examples of GST outcomes illustrated for various clinical trial scenarios

**Figure 1:**
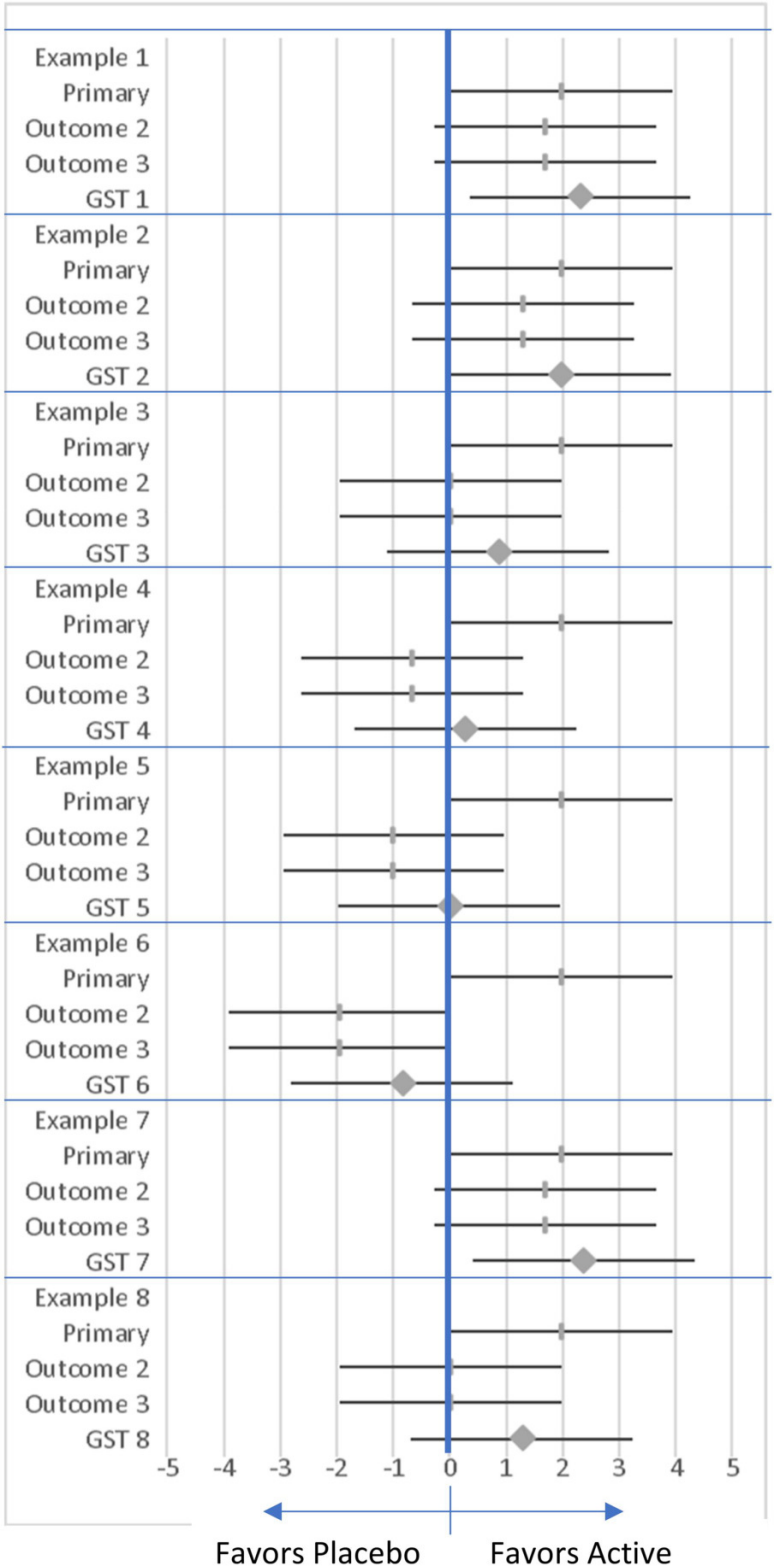
Statistical evidence forest plot of example scenarios with significant primary outcome and two additional outcomes providing different levels of consistency with the primary outcome.

Table 1 and Figure 1 summarize the hypothetical examples described in the methods section. These results are calculated mathematically. Figure 1 is a *Statistical Evidence Forest Plot. P*-values are shown in a tabular format in Table 1. The forest plot is a visual depiction of the strength of evidence present in a given scenario as represented by a z-score test statistic with a 95% confidence interval for a z-score (i.e., +/- 1.96). Since this display emphasizes the strength of evidence but not clinical interpretation of the results, this forest plot could be paired with a more traditional forest plot or the addition of a column with the relevant effect sizes of each outcome.

- Example 1 shows results on the secondary endpoints that are not significant but are supportive of the primary endpoint results and strengthens the overall evidence for a treatment effect. This leads to a GST that is further right than any of the individual outcomes.
- Example 2 illustrates a scenario in which the secondary endpoints are not supportive nor contradictory and the GST *p*-value is similar to the *p*-value for the primary endpoint.
- Example 3 shows that null treatment effects (p∼=0.5 1-sided) on the secondary endpoints substantially weaken a significant result on the primary outcome, since the GST estimate is further left than the primary result.
- Example 4 shows that when the results on the secondary endpoints are directionally opposite of the primary endpoint, the integrated evidence of efficacy is substantially less than the primary result.
- Example 5 shows that for the results of the secondary endpoints to entirely cancel out a significant primary endpoint result, resulting in a neutral GST, they must be in the opposite direction of the primary endpoint with a magnitude sufficient to balance the strength of the primary outcome.
- Example 6 shows that 2 secondary endpoints that are statistically significant in favor of placebo shift the GST estimate to the left of the plot, indicating an overall effect that favors Placebo.
- Example 7 is identical to Example 1, but weights both secondary endpoints half as much as the primary endpoint to reflect greater importance of the primary endpoint. This is done by applying weights to the GST formula as follows:
  $$\mathrm{GST}=\frac{\sum_{k}w_{k}z_{k}}{\sqrt{\sum_{k}w_{k}^{2}+\sum_{k}\sum_{l\neq k}w_{k}w_{l}{\hat{r}}_{kl}\;}}.$$

**Table 1:** Examples of GST *p*-values from 8 hypothetical scenarios.

	2-sided				1-sided			
	Primary Outcome	Outcome 2	Outcome 3	GST *p*-value	Primary Outcome	Outcome 2	Outcome 3	GST *p*-value
Example 1	0.049	0.090	0.090	0.021	0.025	0.045	0.045	0.011
Example 2	0.049	0.196	0.196	0.050	0.025	0.098	0.098	0.025
Example 3	0.049	0.990	0.990	0.391	0.025	0.495	0.495	0.195
Example 4	0.049	0.500	0.500	0.790	0.025	0.750	0.750	0.395
Example 5	0.049	0.320	0.320	0.993	0.025	0.840	0.840	0.503
Example 6	0.049	0.050	0.050	0.401	0.025	0.975	0.975	0.799
Examples with full weight for Primary and half weight for Outcome 2 and Outcome 3								
Example 7	0.049	0.090	0.090	0.018	0.025	0.045	0.045	0.009
Example 8	0.049	0.990	0.990	0.201	0.025	0.495	0.495	0.100

In the case of this example, the 0.5, 0.25, and 0.25 are used for $w_{1}$, $w_{2}$, and $w_{3}$. When the weights are equal and the same correlation is used for each pair of outcomes, this equation simplifies to the GST formula specified earlier.

- Example 8 is identical to Example 3, but weights both secondary endpoints half as much as the primary endpoint to reflect greater importance of the primary endpoint.

These examples clarify an important point: while some may mistakenly believe that GSTs may generate statistical significance of clinically meaningless results, GSTs do not universally increase statistical significance. In fact, the more objective and precise outcomes stemming from the broader scope of evaluation from GSTs fosters more confidence, and therefore more consistent interpretations of the data, regardless of whether that interpretation is a treatment has no benefit, has a small and clinically irrelevant benefit, or has meaningful benefit.

## Simulation results

### No correlation scenario

In the extreme case of zero correlation between outcomes, the true alpha level for a co-primary analysis could be calculated by multiplying the one-sided alpha levels from each test (e.g., 0.025×0.025=0.000625). In a study powered at 80% for a single endpoint, a co-primary analysis would have the power of only 64% for the same effect size on both endpoints.

### Highly correlated outcomes (r = 0.9)

**Figure 2:**
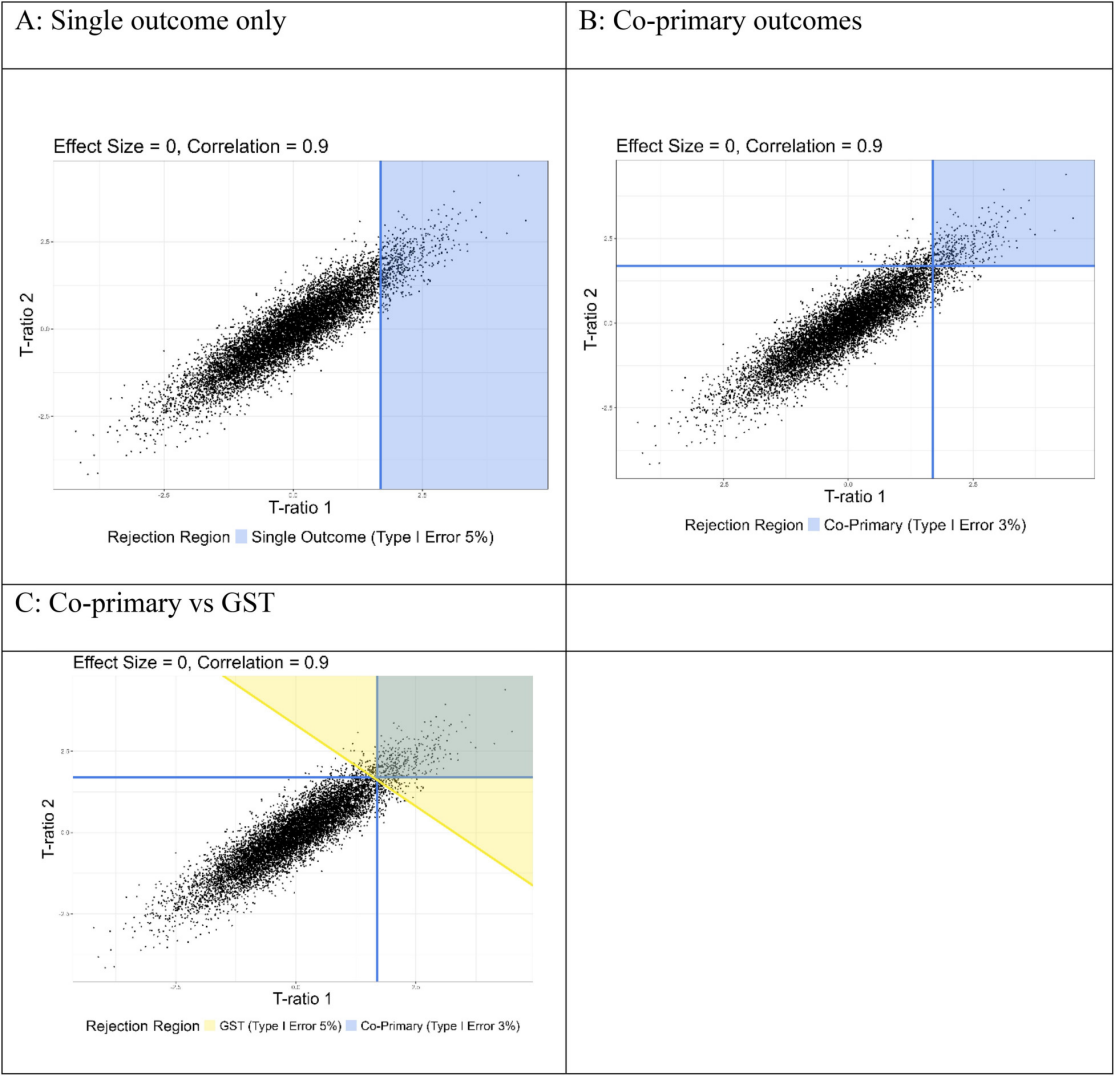
Distribution of simulated outcomes with no treatment effect on either outcome (including both treatment periods from the crossover design, r = 0.9).

Simulation study results were used to examine the effect of correlation on outcomes. First, we examined the spread of our simulated data when a significant result was required on a single primary outcome only (*x*-axis). As expected, with no effect, the T-ratios were symmetrically distributed around the zero value, with a compressed distribution cloud, indicative of a high correlation (0.9; Figure 2A) with the other outcome (*y*-axis). With a 2-sided alpha of 0.10, there was a 5% probability of declaring a significant treatment benefit by chance, which corresponds with the points in the blue-shaded area.

When a significant result was required for co-primary endpoints, the type I error was below 5%, corresponding with stricter standards for declaring significance (Figure 2B). In the null scenario, the proportion of significant outcomes declared by chance using GST methodology was equal to the proportion expected with a single primary outcome (Figure 2C, yellow-shaded area). The yellow-shaded region in each of the figures is representative of the region where a GST returns a significant *p*-value and is calculated linearly from the two t-ratios using the GST formula defined in the methods section.

**Figure 3:**
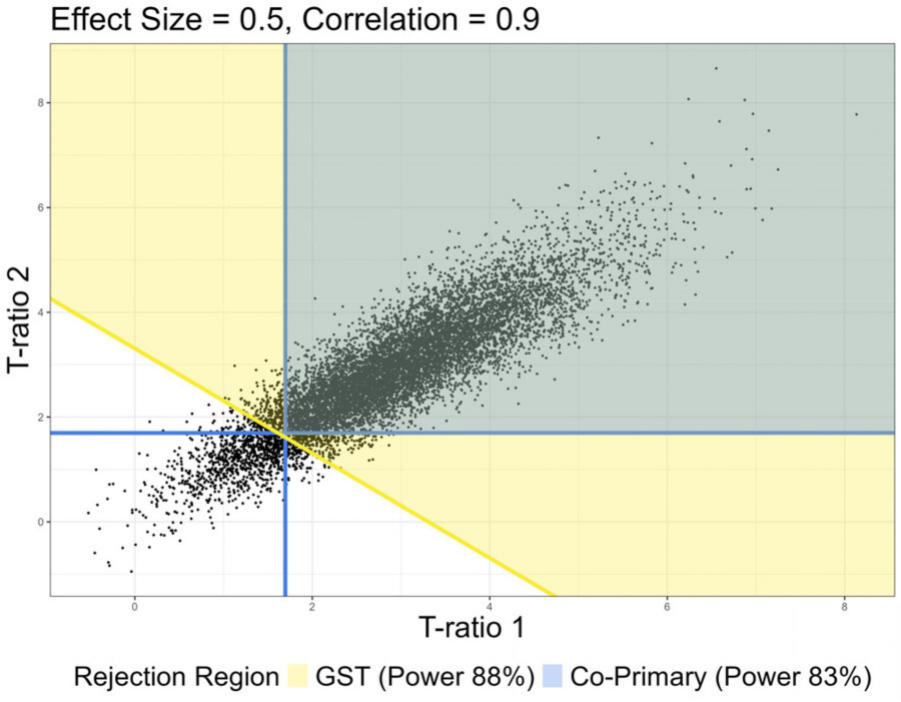
Distribution of simulated outcomes with strong treatment effect on both outcomes (including both treatment periods from the crossover design, r = 0.9).

With a true and similar effect on both outcomes (Cohen's *d* = 0.5), the distribution of simulated datasets shifted up and to the right from the origin. With highly correlated outcomes, most of the endpoints considered significant in GST were also significant in the co-primary analysis (Figure 3), so there was a 5% difference in power (88% vs. 83%) and little difference in type I error (0.05–0.03) (Table 2).

**Table 2:** Power and type 1 error (alpha) for co-primary and GST analysis, by correlation level (including both treatment periods from the crossover design, Cohen's d = 0.5 for both outcomes).

Correlation (r)		Co-primary endpoints	GST
**0.9**	**Power**	83%	88%
	***α***	3%	5%
**0.4**	**Power**	77%	95%
	**α**	0.7%	5%

Bold values are used to distinguish row and column labels from the power and type I error calculations.

### Moderately correlated outcomes (r = 0.4)

**Figure 4:**
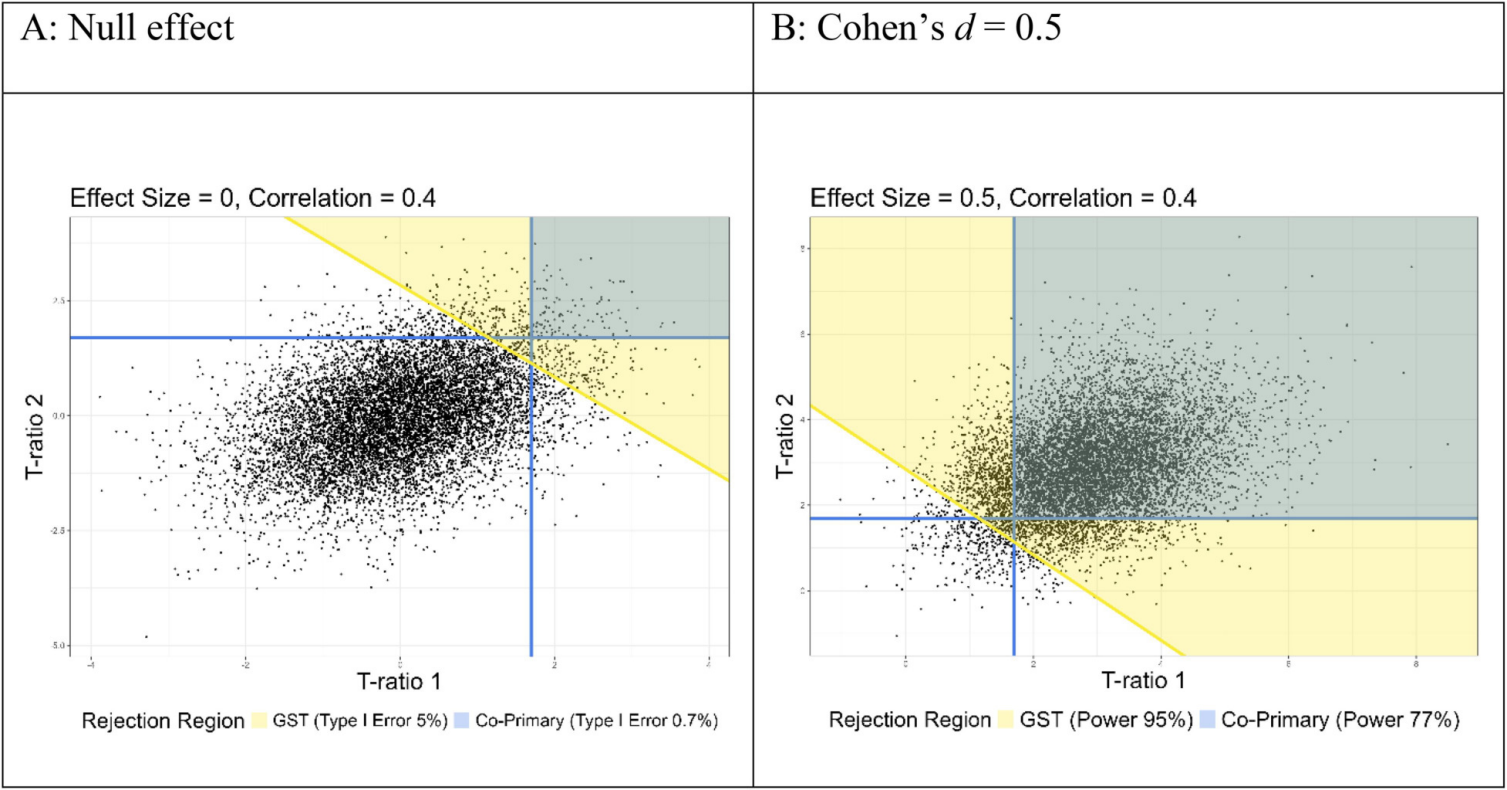
Distribution of simulated outcomes with moderate correlation (including both treatment periods from the crossover design, r = 0.4).

In this scenario, the distribution cloud was notably more dispersed, resulting in substantially more inconsistencies between the two methods (Figure 4A), even when both had a relatively strong true treatment effect (Figure 4B).

The co-primary analysis over-controlled type I error at 0.7% instead of the expected 5%, resulting in an 18 percentage-point decrease in power compared to the GST power, while the GST appropriately controlled type I error at 5% (see Table 2).

### High correlation, true treatment effect on a single outcome only

**Figure 5:**
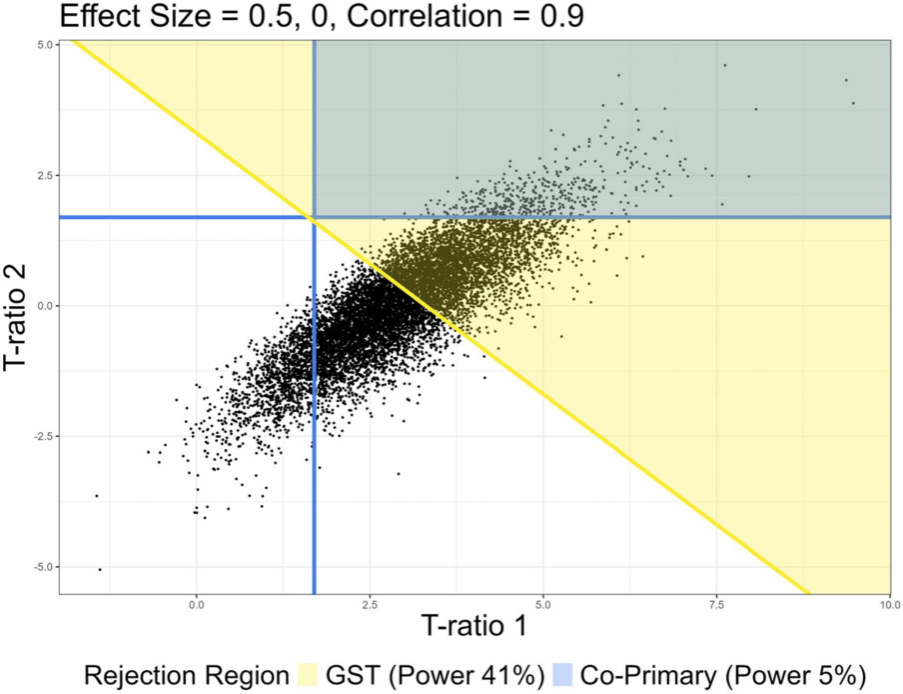
Distribution of simulated outcomes with treatment effect on a single outcome only ((including both treatment periods from the crossover design, r = 0.9).

With only a single outcome showing a true effect, and with a Cohen's *d* of 0.5, we would reject null hypothesis, in favor of treatment benefit, 87% of the time (Figure 5). For the other outcome, with zero effect, the null hypothesis in favor of treatment benefit would be rejected 5% of the time, according to the two-sided alpha of 0.10. A coprimary analysis would consider such a trial a success in 5% of simulated datasets, compared with 41% of datasets in the GST analysis (Figure 5).

### Moderate correlation, varied treatment effects

In simulations in which treatment effects differed between the outcomes, power for the co-primary analysis was consistently lower than the power for the individual outcome with a lower effect size, whereas with the GST, the power was either preserved or increased (Table 3). In this table, Outcome 1 has the higher effect size, though the results hold when effect size is reversed without loss of generality.

**Table 3:** Power at various effect sizes in the cross-over design (r = 0.4).

Effect size (Cohen's *d*)	Power				
Outcome 1	Outcome 2	Outcome 1	Outcome 2	Co-primary	GST
0.5	0.4	86%	72%	66%	91%
0.5	0.3	87%	51%	47%	85%
0.4	0.4	72%	72%	56%	85%
0.4	0.3	72%	50%	41%	75%

### Period 1 only analysis: moderate correlation, varied treatment effects

When comparing just the first treatment periods of the two sequences (analyzed via t-test), the pattern persists of power reduction with the co-primary analysis and preservation or increase of power when using GST (Table 4). Of note, power in these scenarios was lower overall because there were half the observations.

**Table 4:** Power at different effect sizes (first treatment period only, r = 0.4). GST, relationship between outcome correlation and *p-*value*.*

Effect size (Cohen's *d*)	Power				
Outcome 1	Outcome 2	Outcome 1	Outcome 2	Co-primary	GST
0.5	0.5	40%	41%	22%	51%
0.5	0.4	39%	30%	17%	44%
0.5	0.3	40%	21%	13%	37%
0.4	0.4	30%	29%	14%	37%
0.4	0.3	29%	21%	10%	31%

**Figure 6:**
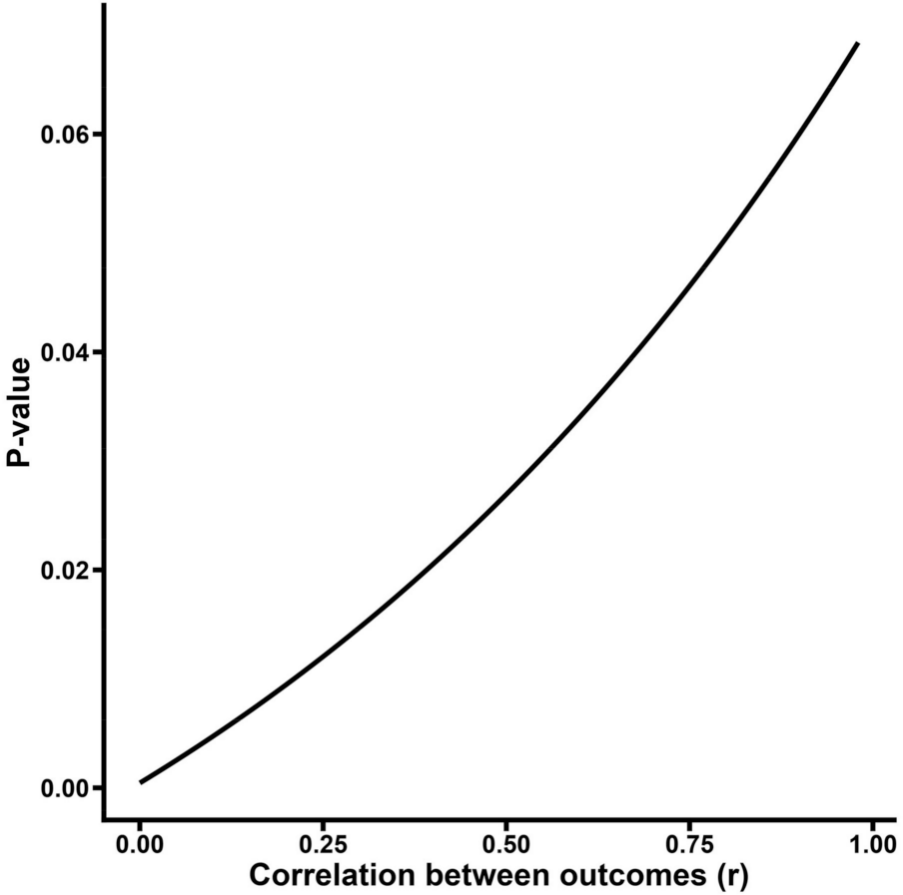
Impact of outcome correlation on statistical testing using the GST.

Varying the levels of correlation between the outcomes changes the amount of unique information each outcome contributes to the GST, which affects the *p*-value calculated in a statistical test. For example, if the *p*-value calculated separately for each outcome is 0.07, the *p*-values calculated using GST would range from 0.0681 for a nearly perfect correlation (r = 0.98) to 0.017 when correlation is zero (Figure 6). In other words, the GST provides more information, and therefore (all else equal) lower *p* values when the correlation is lower, thereby indicating greater independence of outcomes.

**Figure 7:**
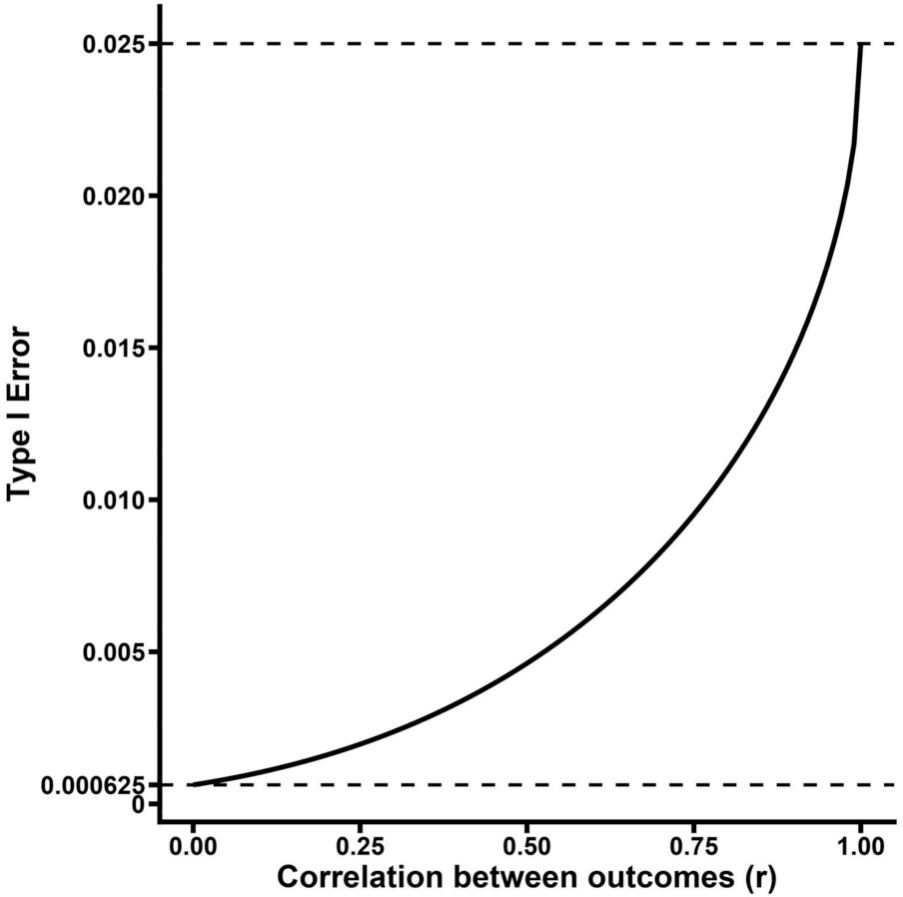
Impact of outcome correlation on type 1 error for co-primaries.

In contrast, co-primary endpoint analyses do not consider the impact of correlation on the amount of information. Requiring multiple co-primary outcomes requires a much stricter threshold for statistical significance, particularly when those outcomes are not highly correlated. In rare disease research, where low correlation between endpoints is common, this approach results in an excessively conservative ‘true’ alpha. For a nominal one-sided alpha = 0.025, the actual type I error rate is only maximized at 0.025 if outcomes are perfectly correlated; if they are independent, it can drop as low as 0.000625 (Figure 7). Consequently, a co-primary strategy over-protects the alpha level, creating an unnecessarily high barrier to success in fields where outcomes are already difficult to measure.

## Discussion

In rare and complex diseases, the clinical trial experience may be insufficient to support a clear choice of primary endpoint or to optimize selection of items for a composite scale. Co-primary endpoints are likely not feasible due to limited sample sizes, and co-primary endpoints and GSTs test different hypotheses. Co-primary endpoints test an “AND” hypothesis – success requires statistical significance on endpoint 1 AND endpoint 2. In contrast, GSTs test an “AVERAGE” hypothesis, and this averaging of endpoints synthesizes information across endpoints into a single hypothesis test and single inference such that the GST gives a more accurate representation of the evidence provided by a clinical trial.

In the present research we illustrated via hypothetical examples and simulated data how GSTs can synthesize information across multiple endpoints in individual trials. The hypothetical examples illustrated the flexibility of GSTs in synthesizing information by allowing different weighting for component endpoints, whereas co-primary endpoints place equal weighting on each endpoint. There are no clear rules on when to impose differential weighting, but the flexibility of weighting outcomes to assist in decisions is appealing. Examples of when outcomes might be weighted in a GST include when regulators may specify that the results of one outcome are more meaningful than another or when patient feedback indicates that one assessment measures an aspect of the disease that is more important to them than other outcomes. Additionally, one key advantage of GSTs was illustrated in Examples 1 and 7, where the GST showed stronger evidence of efficacy than any individual outcome, which occurs when there is consistency across the component outcomes of the GST and becomes stronger when there is less correlation between outcomes.

The simulation study explored the relationship between outcome correlation, statistical power, and type I error control when evaluating two study outcomes simultaneously in a GST. In line with previous findings, the GST approach had greater power while maintaining control of type I error at the nominal rate, compared with co-primary analyses, which creates unnecessary barriers to discovering true effects by over-controlling type I error.

There may be cases where a single primary or co-primary outcomes are a better fit than a GST. For example, when a drug targets a single symptom and an outcome has shown to be the best measure of that symptom, a GST would weaken the ability to detect the treatment effect. Because of the steep price in power associated with co-primaries, there are few situations where a sponsor should choose this route, but one scenario might be when seeking multiple label claims, and then only if the treatment would not be marketable with only a single claim, since it may still be possible to pursue multiple claims through a gatekeeping approach. Treatments for rare diseases will almost never fall into these scenarios, however, and will benefit from the greater power associated and reduced risk of using an average effect across multiple relevant outcomes. Likewise, complex diseases do not always have optimized composites available that have gained enough acceptance by regulators to be used feasibly, so GSTs can provide better results at lower sample sizes, particularly for disease-modifying treatments in progressive diseases. Indeed, GSTs will be useful in any scenario in which synthesis of information from multiple outcomes is useful.

Specific scenarios in which GSTs have been usefully applied include Parkinson's disease (PD), rheumatoid arthritis, and stroke (19–21). In all three cases, the underlying hypothesis is that the treatment has an overall impact on the disease, which would be better reflected on a composite scale of multiple outcomes.

The benefits of GSTs should be considered in light of some limitations. Because the GST combines results from individual outcomes, any bias or violation of assumptions for the individual analysis will also be present in the GSTs constructed from those individual outcomes. However, this limitation also applies to co-primary endpoints. Determining the clinical meaningfulness of GSTs can be accomplished using similar methods to those applied to any other endpoint. It is also useful to include the results from the individual components of the GST, the clinical meaningfulness of which can be determined as would be done if the component endpoints were used outside the context of a GST. One particularly appealing approach to understand the clinical meaning of GSTs with DMTs in progressive diseases is to convert the magnitude of the difference between treatments (vertical distance in a typical longitudinal graph) into a time component test that describes the time saved (horizontal distance) by mapping the result for the active arm at endpoint to the time point in the control arm at which the same level of mean change was observed (22–24).

It is also important to align the outcomes selected for the GST with the prespecified outcome hierarchy. A GST could replace co-primary endpoints. Alternatively, a GST combining a primary and key secondary endpoint would be reasonable, followed by another GST, combining primary, key secondary, and other secondary endpoints. Combining primary endpoints across studies could also be a useful GST application, even with primary endpoints that arose from potentially different analytic approaches.

Finally, given the characteristics of co-primary endpoints highlighted in these results, sponsors and regulators alike need to recognize that trials with co-primary endpoints have vastly different and even punitive operating characteristics compared to trials with single primary endpoints, and accommodations should be made for this fact or they should not be required. For instance, alpha can be increased for the components of a co-primary endpoint so that the overall type I error is controlled at a level similar to other trials. Alternatively, a trial with co-primary endpoints could be considered as containing more information than a single trial alone so that the requirement of two adequate and well-controlled trials could be relaxed accordingly. Recently, researchers from the FDA Office of Biostatistics have proposed that nonparametric global test methods may be more sensitive and efficient for rare diseases with heterogeneous manifestations where a single primary endpoint is impractical (25). This shows increasing awareness of the challenges to rare disease research discussed in this paper, and the potential for global testing methods to be used in future clinical trials. Additionally, other methods that address a global evidence hypothesis like win ratio and combined analysis of function and survival (CAFS) that would also be more effective than co-primary endpoints. Future work could compare these to understand the scenarios in which each performs best.

## Conclusion

Using GST as a prespecified endpoint is appropriate in trials where a clear primary endpoint has not been identified, sample sizes are insufficient to support multiple primary endpoints, or more comprehensive assessments across multiple endpoints are needed to fully evaluate outcomes.

## Data Availability

The original contributions presented in the study are included in the article/Supplementary Material, further inquiries can be directed to the corresponding author.

## Appendix

The R code (R v4.3.0) for calculating the meta-GST for the simulation study:

library(dplyr)

analysis_data |>

mutate(GST = rowMeans[select(., tratio1, tratio2)]/sqrt{[1 + (k-1)*p]/k})

Where:

tratio1 = test statistic from outcome 1

tratio2 = test statistic from outcome 2

k = number of outcomes (2)

*p* = correlation used (0.4 or 0.9)
